# Clinical Neuropathology image 1-2015: Crystal-storing histiocytosis of the central nervous system

**DOI:** 10.5414/NP300847

**Published:** 2014-12-19

**Authors:** Adelheid Woehrer, Gabor G. Kovacs

**Keywords:** crystal storing histiocytosis, brain tumor, lymphoma

## Abstract

Not available.

Crystalloid inclusions are rarely encountered within the central nervous system (CNS). Aside from the more common Hirano bodies and Rosenthal fibers, which occur within neurons and astrocytic processes, both being composed of cytoskeletal proteins, eosinophilic crystalloid inclusions have been recently described within oligodendrocytes in a case of adult-onset, complicated form of hereditary spastic paraplegia [1]. However, crystalloid inclusions may also accumulate within macrophages referred to as crystal storing histiocytosis (CSH). CSH is a rare condition, which most often occurs within the setting of lymphoproliferative disorders, in which immunoglobulins aggregate within the cytoplasm of macrophages [2]. Localized and generalized forms of CSH are distinguished based on the extent of organ involvement [3]. While the lymphoreticular system, bone marrow, kidney, and lung are most often affected, CNS presentation is extremely rare with 5 cases reported in the literature, so far [2, 3, 4, 5, 6, 7]. 

Herein, we add another case of a 56-year-old male patient, who presented with an intra-cerebral tumor-like lesion. After a first stereotactic biopsy, which displayed only reactive changes including some unspecific infiltration by macrophages, more extensive surgery was performed 8 months later. Upon histology, large collections of pleomorphic cells with intracytoplasmic fibrillar and crystalloid inclusions (Figure 1A, B) were apparent. The immunoprofile with prominent reactivity for macrophage-associated CD68 (Figure 1C) and lack of glial fibrillary acidic protein (Figure 1D) suggested the diagnosis of CSH. No previous history of lymphoproliferative disease was stated in this patient. He was then referred to and treated at a foreign tertiary care center without available clinical follow-up. 

In contrast to the previous cases of CNS CSH, which uniformly presented in young females, this is the first case in a middle-aged male patient. All cases described so far share their presentation and restriction to the CNS (localized form of CSH). Potential etiology and immunoglobulin composition of CNS CSH are discussed in detail by Orr et al [7]. Given the hitherto limited clinical experience with CNS CSH, the optimal treatment and prognosis remain to be defined. However, based on 4 patients with clinical follow-up [3, 4, 5, 6, 7], a rather indolent disease course with stable disease following treatment of the underlying lymphoproliferative disorder has been suggested [7]. Hence, recognition of CNS CSH is important, as it warrants screening for an occult but treatable underlying lymphoplasmacytic disorder. 

## Conflict of interest 

The authors declare no conflict of interest. 

**Figure 1:**
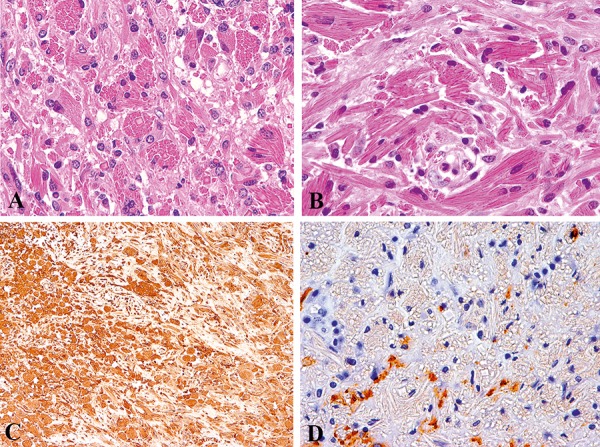
Dense collections of large cells with abundant eosinophilic cytoplasmic inclusions of fibrillar to crystalloid shape (A, B; HE 20× and 40×). Immunohistochemistry reveals their phagocytic lineage with strong expression of CD68 (C, 20×), whereas they are largely negative for GFAP (D, 20×).
